# Characterization of chromatin regulators identified prognosis and heterogeneity in hepatocellular carcinoma

**DOI:** 10.3389/fonc.2022.1002781

**Published:** 2022-09-09

**Authors:** Yin-wei Dai, Han-bin Chen, Ya-ting Pan, Lin-xi Lv, Wei-ming Wang, Xiao-Hu Chen, Xiang Zhou

**Affiliations:** ^1^ Department of Breast Surgery, The First Affiliated Hospital of Wenzhou Medical University, Wenzhou, China; ^2^ Department of Oncology, The First Affiliated Hospital of Wenzhou Medical University, Wenzhou, China; ^3^ Department of Gastroenterology, The First Affiliated Hospital of Wenzhou Medical University, Wenzhou, China; ^4^ Wenzhou Medical University, Wenzhou, China; ^5^ Department of Hepatopancreatobiliary Surgery, The First Affiliated Hospital of Wenzhou Medical University, Wenzhou, China; ^6^ Department of Pathology, The First Affiliated Hospital of Wenzhou Medical University, Wenzhou, China

**Keywords:** chromatin regulator, heterogeneity, metabolism, cuproptosis, hepatoma

## Abstract

Liver carcinogenesis is a multiprocess that involves complicated interactions between genetics, epigenetics, and transcriptomic alterations. Aberrant chromatin regulator (CR) expressions, which are vital regulatory epigenetics, have been found to be associated with multiple biological processes. Nevertheless, the impression of CRs on tumor microenvironment remodeling and hepatocellular carcinoma (HCC) prognosis remains obscure. Thus, this study aimed to systematically analyze CR-related patterns and their correlation with genomic features, metabolism, cuproptosis activity, and clinicopathological features of patients with HCC in The Cancer Genome Atlas, International Cancer Genome Consortium-LIRI-JP cohort, and GSE14520 that utilized unsupervised consensus clustering. Three CR-related patterns were recognized, and the CRs phenotype-related gene signature (CRsscore) was developed using the least absolute shrinkage and selection operator-Cox regression and multivariate Cox algorithms to represent the individual CR-related pattern. Additionally, the CRsscore was an independent prognostic index that served as a fine predictor for energy metabolism and cuproptosis activity in HCC. Accordingly, describing a wide landscape of CR characteristics may assist us to illustrate the sealed association between epigenetics, energy metabolism, and cuproptosis activity. This study may discern new tumor therapeutic targets and exploit personalized therapy for patients.

## Introduction

Hepatocellular carcinoma (HCC) is the most frequent type of primary liver tumor, accounting for > 80% of all liver cancers (1). HCC features include significant inter- and intratumoral heterogeneities (2). A growing body of evidence has elucidated that liver carcinogenesis is a multiprocess that involves complicated interactions between genetic, epigenetic, and transcriptomic alterations. Surprisingly, epigenetic regulation is among the most common abnormal pathways and may contribute to remarkable gene expression changes to accelerate HCC onset and development (3). Chromatin regulators (CRs) are vital regulatory epigenetic factors (4). However, CRs can be classified into three major categories: chromatin remodelers, histone modifiers, and DNA methylators (5–7).

The current studies suggest that aberrant CR expressions are associated with multiple biological processes, including immune activity, apoptosis (8), inflammation (9), proliferation (10), and autophagy (11), which indicates that CR deregulation could lead to poor outcomes in patients with cancer. Epigenetic silencing by SET domain bifurcated histone lysine methyltransferase 1 suppresses tumor intrinsic immunogenicity (12). Of note, the invertibility of epigenetic events makes the epigenetic mechanism an interesting target for therapeutic measures (13).

Altered metabolism is a hallmark of cancer (14–16). Malignant cells are generally known to exhibit nutritional distinctions in comparison with normal cells (17), and accumulating evidence advocates that they also harbor epigenetic changes driven by their rewired cellular metabolism (18–21). In particular, pyruvate kinase directly regulates transcription through histone phosphorylation and chromatin modifier interaction, and a series of chromatin structure changes are mediated by chromatin remodelers under the control of ATP (21).

To our surprise, previous studies have revealed that aberrant chromatin is stronge associated with many cell death pathways, such as Programmed cell death (22), NETosis (23), caspase-dependent regulated necrosis (24), Apoptosis and necrosis (25). However, “Cuproptosis” is a new concept in research (26, 27). Copper-dependent regulated cell death relies on mitochondrial respiration, and copper leads to cell death *via* the direct bonding of copper to lipoylated tricarboxylic acid cycle constituents (26). Illuminating the cuproptosis mechanism might help discern new tumor therapeutic targets and exploit personalized therapy for patients. However, the exact role of cuproptosis in liver cancer remains controversial. This study aimed to explore the characteristics of cuproptosis activity among CRsclusters for the first time.

In the past several years, the fast enhancement of intrinsic mechanism comprehension of HCC development and occurrence has been witnessed. Several diverse molecular subtypes, which are similar to the native biology of HCC (24–26), have been verified. Collectively, these results indicate that HCC is a more complicated disease than formerly understood.

However, the influence of CR-related genes in HCC has not been elaborated. Therefore, this study aimed to analyze the expression profiles from The Cancer Genome Atlas (TCGA: https://www.cancer.gov/tcga), Gene Expression Omnibus (GEO, https://www.ncbi.nlm.nih.gov/geo/), and the International Cancer Genome Consortium (ICGC: https://dcc.icgc.org/) to explore and conduct an in-depth evaluation of CR signatures in HCC. Here, for the first time, we identified CR-related genes in HCC sample groups with different immune cell infiltration features and metabolic and cuproptosis characteristics. Additionally, a CRsscore was constructed to quantify the CR-related pattern in individuals. The CRsscore was developed as a significant independent prognostic index in HCC and had the potential to direct personalized HCC treatment.

## Methods

### Raw data and preprocessing

Comprehensive computerized searches of three publicly available datasets were conducted to procure the messenger RNA (mRNA) expression profiles. The TCGA LIHC cohort (28) included 370 patients and the ICGC LIRI-JP cohort included 232 samples (29). The microarray datasets, including 225 samples of GSE14520, were downloaded from the GEO. A total of 870 CRs were retrieved from previous research (4). The “limma” R package was utilized to select the CRs related to differentially expressed genes (DEGs) between nontumor and tumor tissues in the TCGA LIHC cohort, with a *P-value* of<0.05 and |log2FC| of ≥0.2.

### Weighted gene co-expression network analysis and their modules

WGCNA was applied for pinpointing the HCC clinical characteristic-specific module by running the R package “WGCNA” (30, 31). The expression profiles of CR-related DEGs were utilized as an import for the WGCNA, and clinical characteristics were analyzed and defined as the sample phenotype. The power of β = 10 and scale-free R2 of 0.95 was instituted as the soft threshold parameters to ensure a signed scale-free co-expression gene network. Correlations were calculated between the module eigengenes and clinical information based on the eigengenes function. Several hub genes were considered functionally significant because they were markedly interconnected with nodes in a module. Our study selected an attractive module and identified hub genes by clinical trait significance and module connectivity.

A co-expression network based on the selected module was constructed by the exportNetworkToCytoscape function in the WGCNA R package and visualized in Cytoscape software to obtain hub nodes (32). Hub genes were calculated by applying the cytoHubba plugin based on the maximal clique centrality (MCC) algorithm (33).

### Distinguishing CR-related patterns

A consensus clustering algorithm was applied based on the hub genes to confirm the number of clusters in the TCGA cohort and further validated in the ICGC-LIRI-JP cohort and GSE14520. This step was run and repeated 1,000 times in R using the package ConsensusClusterPlus to guarantee classification stability.

### Assessment of infiltrating immune cells in the tumor microenvironment

A single sample gene set enrichment analysis (ssGSEA) was used to disclose the relative amount of infiltration of 28 immune cells in the TME according to the TCGA-HCC dataset (34). The marker gene sets for TME infiltration of immune cell types were procured from Charoentong et al. (35). The content of immune cells in individual samples in the ssGSEA was estimated by utilizing differentially expressed marker genes. Each enrichment score was denoted by the relative content of each immune cell type. Furthermore, the Kruskal–Wallis test was used to analyze the distinctions in immune cell abundance between CR clusters to better comprehend the associations between CR clusters and immune cell infiltration in HCC.

### Annotation and functional enrichment analyses

Gene-annotation enrichment analyses were utilized to explore the differences in biological processes between distinct CR-related patterns through the package clusterProfiler in R (36). The gene sets of h.all.v7.5.1.symbols were procured from the Molecular Signatures Database v5.1 (MSigDB) (http://www.broad.mit.edu/gsea/msigdb/). Herein, a distinct energy metabolic scoring system was defined based on Dr. Yu et al.’s energy metabolism classifier for breast cancer (36) and the gene sets of HALLMARK_GLYCOLYSIS.v7.5.1, which was acquired from the MSigDB.

Additionally, 10 cuproptosis-related genes were retrieved from the literature and divided into activated and inhibitor groups (**Supplement Table 3**) (26). Moreover, these analyses were run by implementing the ComplexHeatmap and gene set variation analysis (GSVA) (37) packages in R to quantify the heterogeneity in different CR-related HCC patterns.

### Construction of CR phenotype-related gene signature

A scoring system, named CRsscore, was constructed to assess the epigenetic regulation pattern of individuals with HCC as follows. DEGs were identified between CR clusters *via* the package limma in R. The significance criteria for determining DEGs were a *P*-value of<0.001 and |log2 fold change (FC)| of >2.0 (38). DEG intersection from different CR clusters in the TCGA-HCC cohort and the genes involved in the ICGC LIRI-JP cohort were regarded as the ultimate DEGs. The prognostic genes in the TCGA cohort were screened using univariate Cox regression analysis on the premise of correlating (*P*< 0.01) with the overall survival (OS) of patients. All 152 genes were further incorporated into a least absolute shrinkage and selection operator (LASSO) analysis for dimension reduction in the “glmnet” R package. Next, a multivariate Cox analysis further screened five genes based on the lowest Akaike information criterion value. The CRsscore of our model for each sample was determined by the relative expression of each CR phenotype-related gene and its associated Cox coefficient.


$$CRsscore=\;=e^{sum(each\;gene's\;expression\times corresponding\;regression\;coefficient)}$$

The ICGC LIRI-JP cohort was used to validate the prediction effect of the model.

### Drug susceptibility analysis

In order to explore the difference in the responses to chemotherapeutic drugs between the two sets, the semi-inhibitory concentration (IC50) values of the chemotherapeutic drugs which are usually utilized to treat LIHC was calculated by using the “pRRophetic” package.

### Immunohistochemical staining

The tissue microarray (TMA), including 30 paired liver cancer tissues and para-carcinoma tissues, was derived from 2019 to 2021 at the First Affiliated Hospital of Wenzhou University. This study was supported by the hospital’s ethics committee, and all the patients provided informed consent.

TMA sections (4-μm thick) were deparaffinized and hydrated, and 0.3% hydrogen peroxide, and incubated with primary antibody overnight at 4°C and with secondary biotinylated goat anti-rabbit antibody successively; the sections were then stained using SignalStain^®^ DAB (Cell Signaling Technology, Danvers, MA) and counterstained with hematoxylin QS (Vector Laboratories). The intensity of staining (0, 1, 2, 3) and the proportion of positive cells (0%–100%) were semi-quantified, and scored from 0 (no stained cells) to 3 (all cells intensely stained). The detail information of tissue microarrays is available in **Supplementary Table 1**.

### Statistical analysis

Pearson correlation was used to analyze the correlations between variables, and a t-test was used to explore the continuous variables that conformed to a normal distribution between binary groups. The Kruskal–Wallis test was utilized to distinguish the differences for comparison of the three clusters. The cutoff values of each dataset were evaluated with the survival outcome and CRsscore in each dataset using the R package survminer. The Kaplan–Meier method was applied to depict survival curves for the subsets in each cohort, and the log-rank test was used to statistically identify significant differences. Significance was defined at *P*< 0.05 in the premise. All statistical analyses were performed using R, version 4.1.0.

## Results

### Data processing

**Figure 1:**
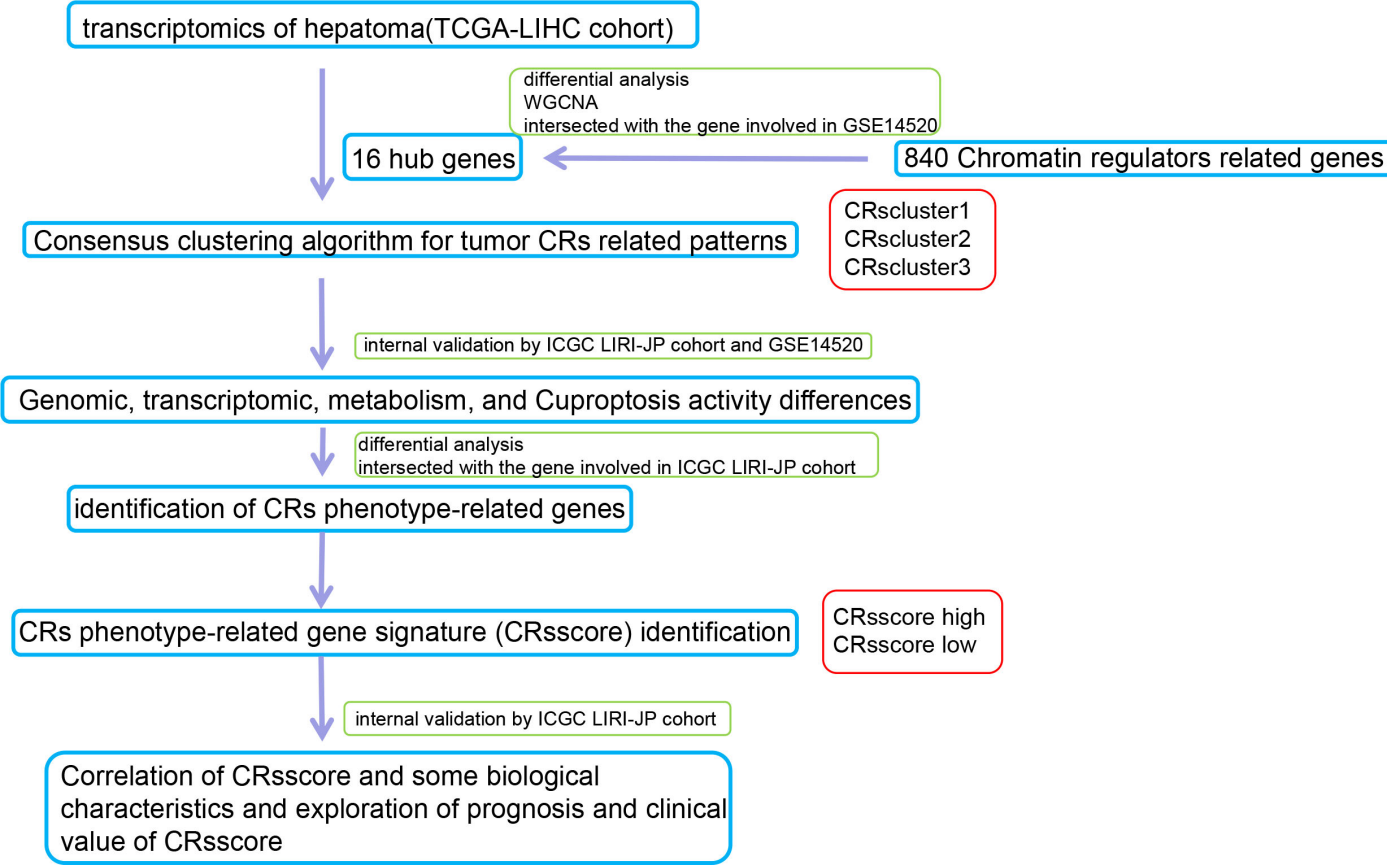
Schematic summary of the workflow.

A flow chart of the data processing and course in this study is presented in **Figure 1**.

### DEG screening

The expression matrix was obtained from the 370 samples in the TCGA cohort after data processing and quality assessment. A total of 549 DEGs (267 upregulated and 282 downregulated) were derived for subsequent analysis under the threshold of a *P*-value of<0.05 and |log2FC| of ≥0.2 (**Supplement Table 2**).

### Co-expression network construction

**Figure 2:**
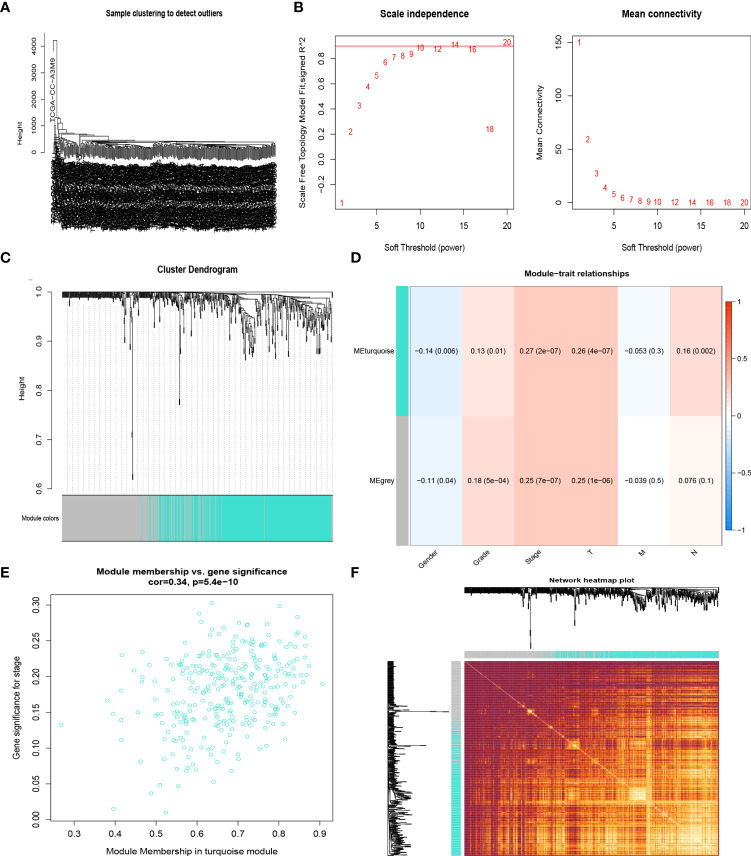
CR-related genes in the TCGA-HCC cohort by the weighted gene co-expression network analysis (WGCNA). Sample dendrogram **(A)** and the mean connectivity and scale independence of the WGCNA analysis **(B)**. Clustering dendrograms of samples in the TCGA-HCC cohort **(C)**. Heatmap of the correlation between module eigengenes and disease progression of HCC **(D)**. Scatter plot of module eigengenes in the turquoise module **(E)**. Heatmap describing the topological overlap matrix among genes based on co-expression modules **(F)**.

The samples of the TCGA LIHC cohort were clustered using the average linkage and Pearson’s correlation methods (**Figure 2A**), and the co-expression network was developed by implementing the co-expression analysis. The power of β = 10 (scale-free R2 = 0.95) in this study was screened as the soft-thresholding parameter to guarantee a scale-free network (**Figures 2B, C**). In total, two modules were found by the average linkage hierarchical clustering. The turquoise module had the most significant relationship with the tumor stage (**Figures 2D, E**), and this module was screened as the crucial clinical module for further exploration. Finally, the association between different modules was illustrated by an eigengene adjacency heatmap (**Figure 2F**).

### RF-related classifier identification and validation

A total of 30 hub genes were calculated by applying the cytoHubba plugin based on the MCC algorithm (33)(**Supplement Figure 1A**). The intersection between the 30 hub genes and the gene involved in GSE14520 was taken to apply this classifier to multiple datasets. Finally, an RF-related classifier involving 16 hub genes was customized (**Supplement Table 3**).

### Three different CR-related patterns identified by unsupervised learning

**Figure 3:**
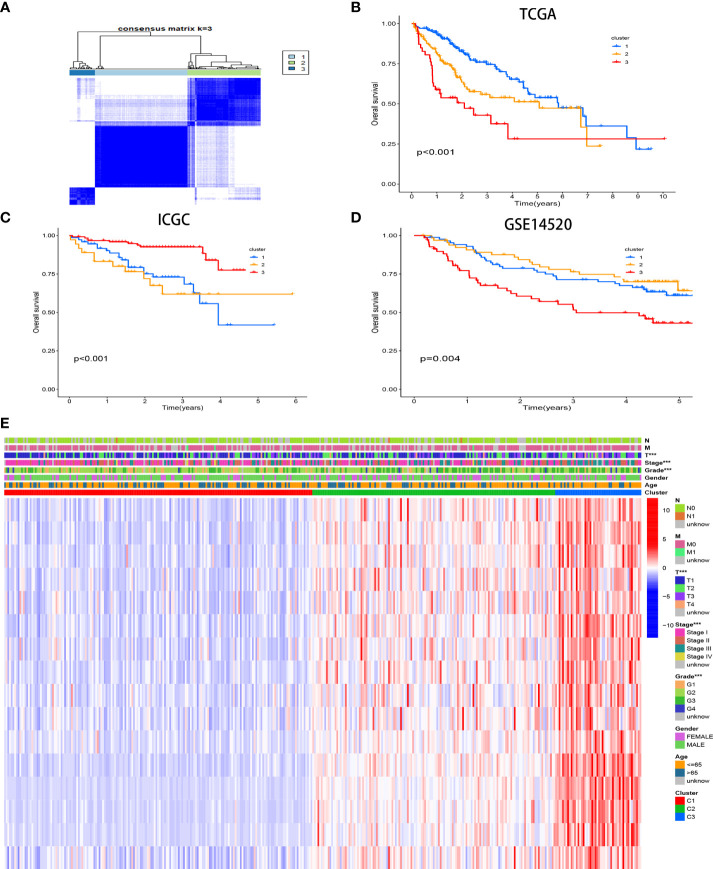
Consensus matrices of samples in the TCGA-HCC cohort *via* the unsupervised consensus clustering method (K-means) **(A)**. Survival analysis of the different CR clusters in the TCGA **(B)** and ICGC **(C)** cohorts and GSE14520 **(D)**. Heatmap of the clinicopathological manifestations among the CR clusters **(E)**.

Three unique CR clusters (**Figure 3A**) were identified by unsupervised clustering in the TCGA cohort according to 16 hub genes. Importantly, analysis from the ICGC LIRI-JP cohort as well as GSE14520 externally verified the stability of our clustering results (**Supplement Figures 2A, B**). The Kaplan–Meier analysis illustrated that cases in cluster 3 correlated with more adverse prognoses (**Figure 3B**). Similar results were obtained for OS in the ICGC LIRI-JP cohort and GSE14520 (**Figures 3C, D**). Hence, the classifier robustness was well validated. The cluster-related gene expression distribution and specific clinical characteristics between the subgroups were revealed (**Figure 3E**). Our CR-based classification revealed the CR-related gene expression levels, which were abundant in cluster 3. Similar results were obtained in the ICGC LIRI-JP cohort as well as GSE14520 (**Supplement Figures 3A, B**). These results draw the same conclusion that patients with HCC with poor outcomes are abundant with the 16 hub gene expressions.

### Levels of infiltrating immune cells in patients with different CR-related subtypes

**Figure 4:**
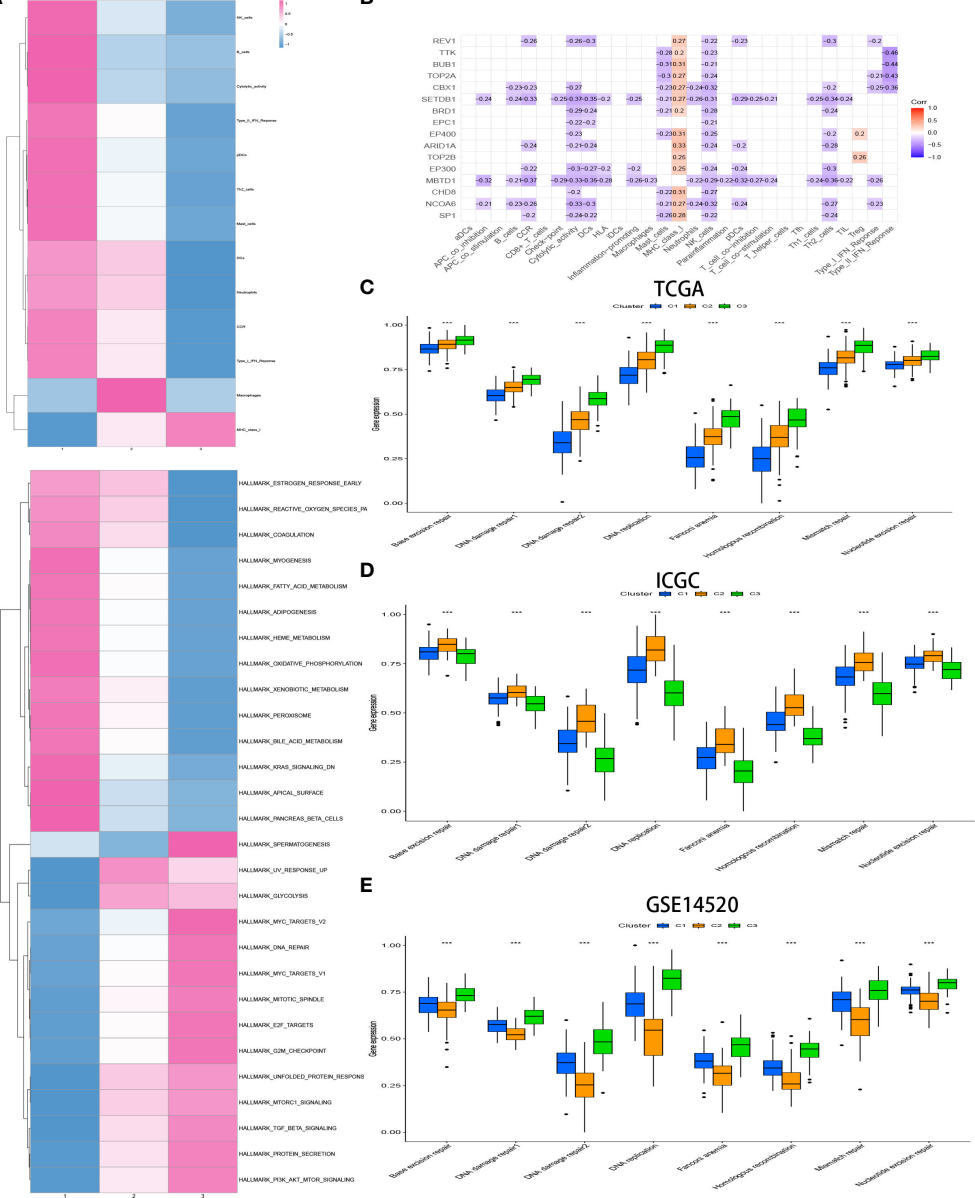
Heatmap of immune responses among the CR clusters **(A)**. Analysis of the hub genes–immune response relationships of HCC based on TCGA data **(B)**. Exploration of the difference in DNA damage repair pathways among CR clusters in the TCGA **(C)** and ICGC **(D)** cohorts and GSE14520 **(E)** by ssGSEA analysis. GSVA enrichment analysis exhibits the activation status of biological pathways among different CR clusters (Kruskal–Wallis test, *P*< 0.05), with red representing activation and blue representing inhibition **(F)**. *p<0.05,**p<0.01,***p<0.001

The relative amount of infiltrating immune cell constitution in the TME of HCC between the CRsclusters was calculated *via* the ssGSEA algorithm to find the correlation between TME and CR-related subtypes. Moreover, a heatmap was utilized to visualize the significant differential levels of infiltrating immune cells, which were defined with a strict cutoff of p< 0.05 by the Kruskal–Wallis test (**Figure 4A**). CRscluster1 had features of high TME immune cell infiltration with conspicuous surges in the infiltration of natural killer cells, B cells, pDCs, Th2 cells, mast cells, DCs, and neutrophils as well as cytolytic activity, tumor necrosis factor (TNF) II interferon (IFN) response, TNF I IFN response, and CCR. **Figure 4B** reveals the surprising negative correlation between the 16 hub gene expression and most immune signatures. This is consistent with our conclusion.

### Features of the biological process in distinct CR-related subtypes

The biological process among CR-related clusters was further explored *via* performing ssGSEA for hallmark gene sets. **Figure 4A** shows that CRscluster1 was strongly activated in stromal and metabolism pathways, such as glutathione, fatty acid, and phenylalanine metabolism pathways. CRscluster3 was markedly related to carcinogenic and DNA damage repair-associated pathways. CRscluster2 was the intermediate state of the other two clusters. These phenomena are similar to the GSEA analysis results (**Supplement Figure 3A**).

Subsequently, markers that represent DNA damage repair signaling pathways were screened (**Supplement Table 4**) and determined among different clusters by ssGSEA. The phenomenon (**Figure 4C**) illustrated that CRscluster3 is associated with better DNA damage repair than the other CRsclusters. The ICGC LIRI-JP cohort and GSE14520 analysis drew the same conclusion (**Figures 4D, E**), considering the survival analysis results (**Figures 3C, D**).

**Figure 5:**
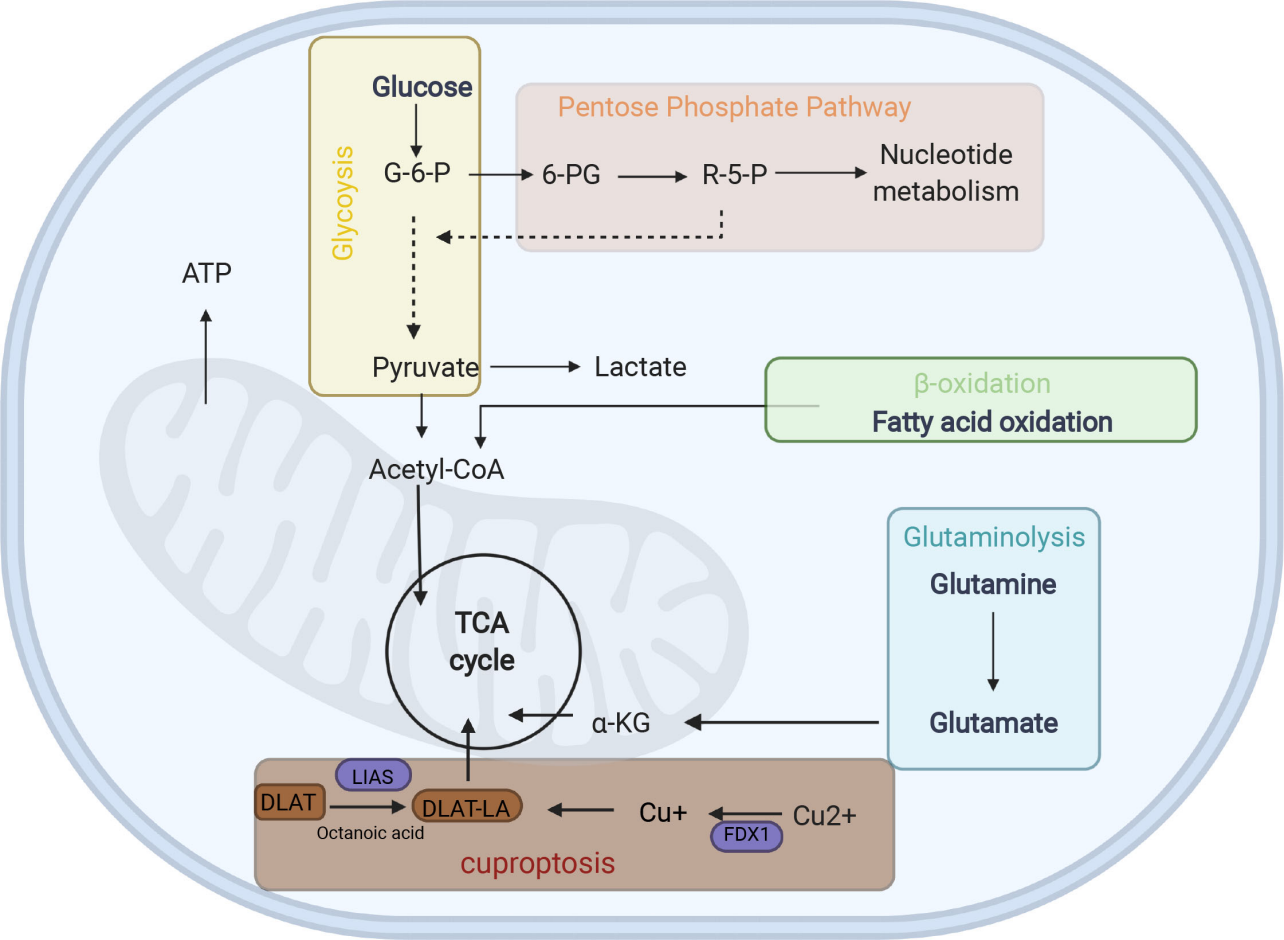
Energy metabolism diagram. G6P glucose-6- phosphate, 6-PG Glucose 6-phosphate, R-5-P ribose-5-phosphate, α-KG α-ketoglutarate, LA lipoylation, DLAT, a protein target of lipoylation.

**Figure 6:**
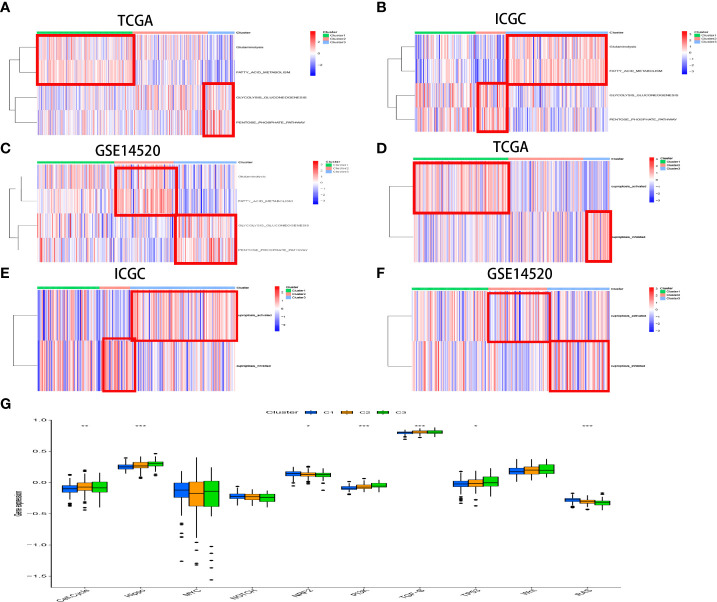
The different activated statuses of metabolism pathways among different CR clusters was exhibited by ssGSEA in the TCGA **(A)** and ICGC **(B)** cohorts and GSE14520 **(C)**. The distinct statuses of cuproptosis activity among different CR clusters was exhibited by ssGSEA in the TCGA **(D)** and ICGC **(E)** cohorts and GSE14520 **(F)**. The boxplot illustrates score variations in 10 vital cancerogenic signaling pathways between the CR clusters **(G)**.

The customized energy metabolic scoring system containing four central metabolic pathways, including glycolysis, glutaminolysis, fatty acid oxidation (FAO), and the pentose phosphate pathway (PPP) (39)(**Figure 5A**, **Supplement Table 4**), was used to further explore the metabolic heterogeneity among different clusters. The abundance of the activities of the four metabolic pathways was then evaluated by ssGSEA among different clusters. As expected, CRscluster1 was more dependent on FAO and glutaminolysis. CRscluster3 was more dependent on glycolysis and PPP. CRscluster2 was the intermediate state of the other two clusters (**Figure 6A**). Surprisingly, the ICGC LIRI-JP cohort and GSE14520 analysis externally verified the robustness of our results (**Figure 6B,C**). Moreover, the relationship between the four metabolic pathway activities and outcomes in this study followed breast cancer (39). We then analyzed the activity of cuproptosis-related genes by ssGSEA among different clusters. CRscluster1 was abundant with cuproptosis activity (**Figure 6D**). The ICGC-LIRI-JP cohort and GSE14520 show similar results in combination with the finding of the survival analysis (**Figures 6E, 6F**).

The enrichment score of 10 classical oncogenic pathways was evaluated *via* referred signatures (**Figure 6G**, **Supplement Table 4**). Oncogenic pathways, such as hippo-related signaling and phosphatidylinositol 3-kinase (PI3K) signaling, had higher scores in CRscluster3. These results are consistent with those of previous studies associated with the glycolytic cancer tendency (39).

### Development of the CR phenotype-related gene signature

CRs exert a profound effect on shaping different TME landscapes, but the CR-related pattern in individuals cannot be conveniently predicted. Hence, we tried to develop a set of CRsscores to quantify the CR-related pattern of individuals with HCC. We first identified 902 DEGs across clusters 1–3 (**Supplement Table 5**). Additionally, we applied a GO and KEGG analysis to explore the biological pathways associated with the DEGs. DEGs between diverse CR phenotype-related patterns were found to be enriched in metabolism and epigenetic-related biological processes (**Supplement Figures 4A, B**).

In order for the CRsscore to be well validation by other datasets, the final 429 DEG2, which intersected between the 902 DEGs and the gene involved in the ICGC LIRI-JP cohort, were further analyzed as the candidates (**Supplement Figure 5A**). The univariate Cox analysis selected 152 CR phenotype-related genes (**Supplement Table 6**) that were incorporated in the LASSO and multivariate Cox analyses. Eventually, five genes (CDCA8, NEIL3, ANXA10, PON1, and CYP26B1) were identified as independent HCC prognosis indicators. Consequently, we developed the CRsscore using the following formula: CRsscore = (0.088144 ´ expression of CDCA8) + (0.18774´ expression of NEIL3) + (-0.023476 ´ expression of ANXA10) + (-0.002680 ´ expression of PON1) + (0.081717 ´ expression of CYP26B1).

**Figure 7:**
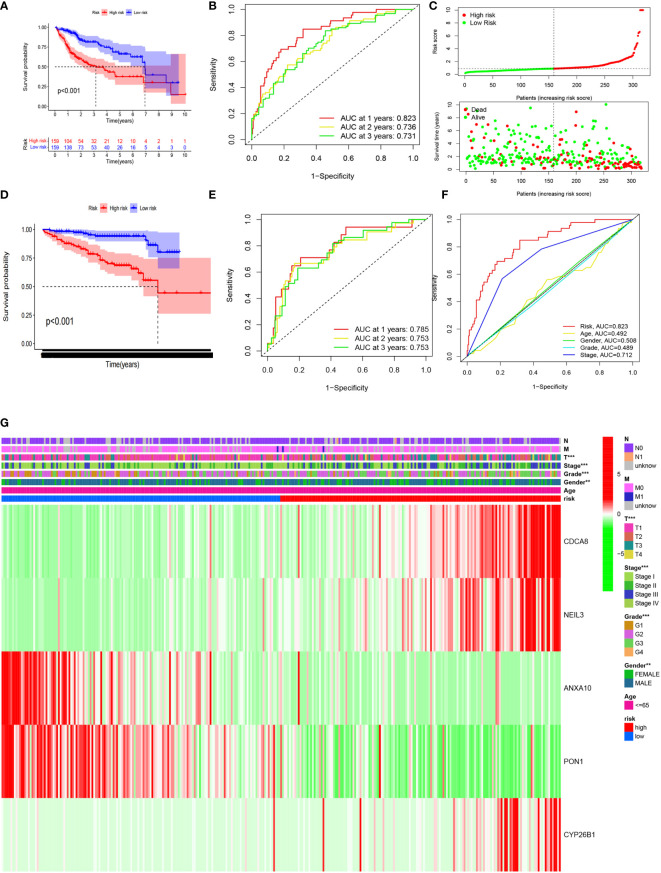
The CRsscore in the TCGA cohort. Kaplan–Meier curves **(A)**, time-dependent ROC analysis **(B)**, risk score **(C)**, and multi-index ROC analysis **(F)**. Thermograph of the clinicopathological features among risk subgroups **(G)**. The CRsscore was validated in the ICGC cohort. Kaplan–Meier curves **(D)** and time-dependent ROC analysis **(E)**.

Samples with HCC in the high CRsscore set had a shorter OS according to the K-M survival analysis (*P*< 0.001, **Figure 7A**). Moreover, the area under the curve (AUC) of the CRsscore was 0.823, which shows a more accurate predictive ability than that of the traditional clinicopathological features (**Figure 7F**). The predictive value of the AUC of the CRsscore regarding the 1-, 2-, and 3-year survival rates was 0.823, 0.736, and 0.731, respectively (**Figure 7B**).

The hazard ratio and 95% CI of the CRsscore in the univariate (*P*< 0.001) and multivariate Cox regression analyses (*P*< 0.001) respectively elucidated the CRsscore as a cancer indicator (**Supplement Figures 5B, C**) and independent prognosis index of OS in patients with HCC.

The heatmap of the association between the clinicopathological features and the CRsscore is also presented (**Figure 7G**). A hybrid nomogram (c-index = 0.735) encompassing the CRsscore and clinicopathological features is shown in **Supplement Figure 5D**. The practical and predicted 1-, 2-, and 3-year survival rates following the reference curve *via* the calibration curve analysis are depicted in **Supplement Figure 5F**. These findings suggest that the nomogram was precise and steady; therefore, its implementation in the clinical services of patients with HCC is appropriate.

The CRsscore of each patient in the ICGC LIRI-JP cohort was also calculated, and the cohort was divided into two groups based on the median value. A survival analysis illustrated a better outcome in the low-risk group (log-rank test; *p*< 0.001; **Figure 7D**). An analysis of the 1-, 2-, and 3-year prognostic prediction classification efficiencies suggested that the CRsscore still had relatively high AUC values (**Figure 7E**), indicating that the CRsscore had a prominent ability to predict HCC prognosis.

### CRsscore was a predictive biomarker for some biological characteristics

**Figure 8:**
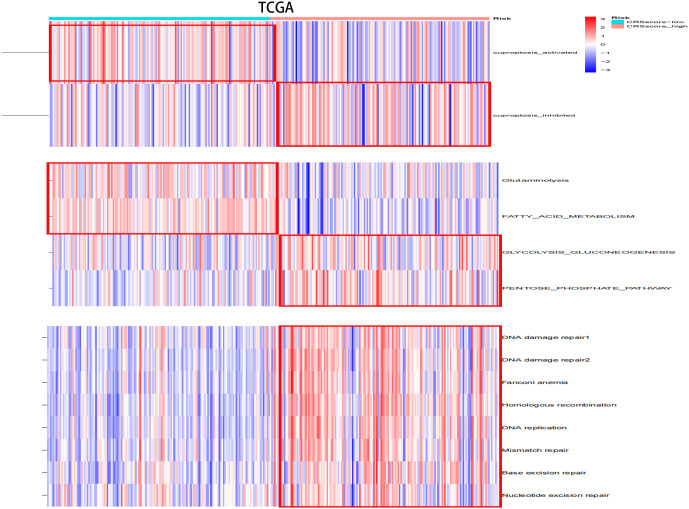
The variation scores of significant biological processes by ssGSEA analysis among risk subgroups in the TCGA cohort.

The energy metabolism level, cuproptosis level, and DNA damage repair pathway were further explored among CRsscore-high and -low groups by applying ssGSEA (**Figure 8A**, **Supplement Figure 4A**). CRsscore-high, which was associated with adverse outcomes, showed more abundant DNA damage repair pathway, glycolysis, and PPP. CRsscore-low, which was related to a benign prognosis, showed more abundant cuproptosis, FAO, and glutaminolysis. These results were identical to the results of the CR cluster analysis. Moreover, **Supplement Figures 7A–E** reveal that the levels of cuproptosis activity, FAO, and glutaminolysis in patients with HCC significantly decreased as the CRSsscore increased. In contrast, the glycolysis and PPP levels increased. Lastly, we further explored the correlation between the CRsscore and cuproptosis activity from a genomics perspective (**Supplement Figure 7F**). Surprisingly, the CRsscore had a significant negative correlation with FDX1 expression, which was the most crucial for cuproptosis regulation (26).

### Chemotherapy sensitivity related to the CRsscore

The IC50 values which can reflect the sensitivity to chemotherapeutic drugs of usual chemotherapeutic drugs were predicted and compared. Patients in the low-CRsscore group were more susceptible to Sorafenib and Gefitinib, whereas patients in the high-CRsscore set were more responsive to Cisplatinl, Mitomycin.C and Doxorubicin(**Supplement Figures 8A–E**).

### Verification of the protein expression of the CRsscore-related molecules

**Figure 9:**
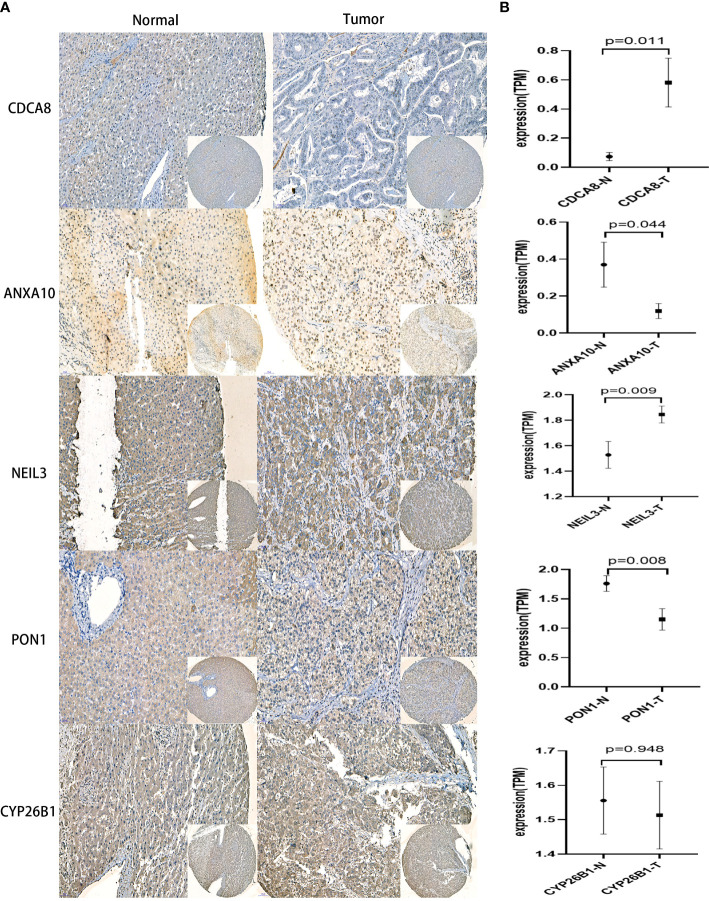
IHC analysis in the First Affiliated Hospital of Wenzhou University cohort. **(A)** Representative IHC staining of CRsscore-related genes in HCC and normal tissues. **(B)** Comparison of the relative expression of CRsscore-related genes between HCC and normal tissues. *p<0.05,**p<0.01,***p<0.001

Immunohistochemical images of CDCA8, NEIL3, ANXA10, PON1, and CYP26B1 were obtained from the First Affiliated Hospital of Wenzhou University cohort(**Figure 9**). These results have indicated that higher expressions of PON1, ANXA10 genes in para cancerous tissues. Meanwhile, higher expressions of CDCA8, NEIL3 genes in liver cancer and the expression of CYP26B1 was no significant difference between para-cancer and cancer. Most important of all, there were many studies involved in the relative expression of PON1 (40–43), ANXA10 (44, 45), CDCA8 (46–48) and NEIL3 (49, 50) in HCC consisting of my experiment. This further revealed validity of the CRsscore.

## Discussion

Previous studies stratified patients with HCC through unsupervised clustering of tumors based on genomics and transcriptomics data because HCC possesses high heterogeneity (51–54). This resulted in the discovery of many patient group-specific distinctions, such as immune responses (52, 53)and metabolism (51), hepatic stem-like phenotypes (54), and cholangiocarcinoma-like traits (55).

To the best of our knowledge, this is the first study to establish a CR-related classifier for several liver cancer cohorts. Our analyses contained genomics, transcriptomics, metabolomics, and clinical data among three data sets with hundreds of HCC tumors. Obvious differences were identified in metabolic signaling pathways, cuproptosis activity, and clinical survival between the three major HCC subtypes. CRscluster1, which was associated with better outcomes, exhibited a higher level of infiltrating immune cells, lesser DNA damage repair pathway ability, more dependence on FAO and glutaminolysis, less dependence on glycolysis and PPP, and more cuproptosis activity. Interestingly, mitochondrial respiration is required for copper-induced cell death (26, 56). CRscluster1 was more abundant in oxidative phosphorylation (**Figure 4F**). This is consistent with our results.

CRscluster3, which was related to poor outcomes, revealed a lower level of infiltrating immune cells, more DNA damage repair pathway ability, less dependence on FAO and glutaminolysis, preference for utilizing glycolysis and PPP for survival, and less cuproptosis activity. Moreover, CRscluster3 tumors were correlated with multiple malignancy characteristics. For instance, the transforming growth factor-β signal pathway, which is related to the hypoxic response, metastasis, malignancy, and Treg cell induction (57, 58), was upregulated in CRscluster3. PI3K/AKT/mTOR signaling activation is one of the crucial features of this tumor group. Asparagine synthetase, PPP, and glycolysis are also activated by PI3K/AKT/mTOR signaling (59), consistent with our observations of CRscluster3. Drugs that target PI3K/AKT/mTOR signaling or processes, such as rapamycin, l-asparaginase, or their analogs, are regarded as potential therapeutics for CRscluster3 treatment but not the other CR clusters.

Warburg (59) concluded that tumor cells tend to utilize glucose for glycolysis despite sufficient oxygen. Tumor proliferation and immune escape were gradually acknowledged to be fueled by aggravated glycolysis (60). Liver cancer cases in CRscluster3, which had a worse prognosis, had higher glycolytic levels and lower oxidative phosphorylation, indicating that CRscluster3 had a strong Warburg effect, and glycolysis does not synergistically act with fatty acids and glutamine to fuel tumor development. Furthermore, the metabolomics analysis revealed the following trend: downstream metabolite accumulation and decreased upstream metabolites. Consequently, diverse therapeutic measures that target metabolic heterogeneities are indispensable. CRscluster2, which correlated with a median outcome, had intermediate performance of the other two clusters. The ICGC LIRI-JP cohort and GSE14520 exploration externally validated the universality of our results in combination with the survival analysis conclusions. Additionally, the CRsscore involved NEIL3, CDCA8, ANXA10, PON1, and CYP26B1 might be acknowledged as an indispensable reference for predicting the outcome of patients with LIHC. NEIL3 is a multifunctional glycosylase. When knocking down NEIL3 in Huh-7 and HepG2 cells, cell abilities including growth, proliferation, invasion and migration, displayed deficiency to different degress (49). NEIL3 can repair Oxidative Lesions at Telomeres during Mitosis in order to avert Senescence in Hepatocellular Carcinoma (50). Meanwhile, CDCA8 knockdown also inhibits cell proliferation and promotes cell differentiation in colorectal cancer, lung cancer, breast cancer, cutaneous melanoma, and human embryonic stem cells (61–65). Besides, ANXA10 is the latest ANXA member (66). In former studies, overexpression of ANXA10 suppresses proliferation and promotes apoptosis of hepatoma (67). ANXA10 boosts melanoma metastasis *via* inhibiting E3 ligase TRIM41-directed PKD1 degradation (68). ANXA10 suppresses papillary thyroid carcinoma apoptosis and promotes proliferation by up-regulating TSG101 thereby activating the MAPK/ERK signaling pathway (69). Moreover, PON1 is a high-density lipoprotein- associated protein. Knockdown of PON1 obviously lessened the cytotoxicity of sorafenib in Huh7 cells (43). Microvascular invasion could be diagnosed depending on serum PON1 (70).​ PON1 was identified to be a potential marker of prognosis in patients with breast cancer recurrence (71).

What’s more, the CRsscore was constructed to estimate and quantify the energy metabolism and cuproptosis activity of individuals with HCC. The low CRsscore group was abundant in glutaminolysis, FAO, and strong cuproptosis. In contrast, the high CRsscore group was abundant in glycolysis and PPP. Notably, these conclusions were well verified in the ICGC LIRI-JP cohort.

Constructing the CRsscore makes it possible to adequately utilize the unique metabolic variations and cuproptosis activity differences in HCC therapy. Targeting glycolytic enzymes in HCC therapy is speculated to be an efficient approach, as some related medicines are now under investigation and will be gradually accepted (72, 73). PPP suppression has been used in cancer therapy apart from glycolysis, and the enzymes symbolic for the non-oxidative or oxidative phase of PPP are transketolase (TKT) and glucose-6-phosphate dehydrogenase, respectively. Both enzymes are upregulated and positively correlated with worse outcomes and aggressive clinicopathological HCC characteristics (74, 75). A study proved that oxythiamine, which is a TKT inhibitor and thiamine antagonist, mechanically suppresses HCC cell growth both *in vitro* and *in vivo* by increasing the reactive oxygen species levels (76). Moreover, glutaminolysis is a significant metabolic characteristic of malignant cells. Glutamine-based therapy has been shown to be useful for cancer treatment (77). Regarding therapy that targets fatty acid metabolism, some studies have proved that TVB-3166 and TVB-2640, fatty acid synthase (FASN) inhibitors, have anti-tumor effects in preclinical colorectal and breast cancer models, as well as limited systemic toxicity and favorable tolerability in early-phase clinical trials (78, 79). Currently, no FASN inhibitors are being tested in clinical trials for HCC treatment. However, FASN inhibitors are used in other cancer types to guide HCC treatment. Notably, a potential link was found between cuproptosis and energy metabolism and epigenetics, which needs to be further tested in the future. This will promote the understanding of cuproptosis activity and metabolism heterogeneity. Personalized management still has a long way to go before it is substantially improved.

This study has some limitations. First, the stability of the CRsscore and CR-related gene classifier was tested and validated in common datasets. The analysis of prospective cohorts would have been more cogent. Second, scRNA-seq, the most advanced technology, should be further combined for future analysis to evaluate possible distinctions in tumor heterogeneity, cuproptosis activity, and intercellular communication between the CRsscore-high and CRsscore-low groups at single-cell resolution. Lastly, we did not illustrate the roles of the genes in HCC at a comprehensive level by experiments when exploring the genes involved in the CRsscore. Therefore, the underlying mechanisms of the genes in HCC should be investigated in the future.

## Conclusion

Overall, comprehensively assessing the CR-related patterns of individuals with HCC using the CRsscore was credible and the CRsscore was related to clinical, cellular, and molecular features, containing clinical stages, energy metabolism, and cuproptosis activity. Furthermore, the CRsscore could be identified as an independent prognostic index for patients with HCC and could roughly evaluate their level of energy metabolism and cuproptosis activity. This adequately utilized the unique metabolic variations in HCC therapy and developed novel target treatment based on cuproptosis activity and chromatin regulators continue to be a huge obstacle for pharmacologists, biologists, and clinicians.

## Data availability statement

The datasets presented in this study can be found in online repositories. The names of the repository/repositories and accession number(s) can be found in the article/**Supplementary Material**.

## Ethics statement

This study was reviewed and approved by the Ethics Committee of the First Affiliated Hospital of Wenzhou Medical University. The patients/participants provided their written informed consent to participate in this study.

## Author contributions

DYW designed the studies and finished most of the work. CHB, CXH completed the experiment, PYT drafted the article. LLX, WWM contributed to data collection and analyses. ZX revised and reviewed the articles. All authors read and approved the final article.

## Funding

This study was supported by the funding of the Wenzhou Science and Technology Plan Project (No. Y20190206).

## Conflict of interest

The authors declare that the research was conducted in the absence of any commercial or financial relationships that could be construed as a potential conflict of interest.

## Publisher’s note

All claims expressed in this article are solely those of the authors and do not necessarily represent those of their affiliated organizations, or those of the publisher, the editors and the reviewers. Any product that may be evaluated in this article, or claim that may be made by its manufacturer, is not guaranteed or endorsed by the publisher.

## Supplementary material

Supplementary Figure 1 The variation scores of significant HYPERLINK "javascript:;" biological HYPERLINK "javascript:;" processes by ssGSEA analysis among risk subgroups in the ICGC cohort.

Supplementary Figure 2 Heatmap of the clinicopathological manifestations among CR clusters in the ICGC cohort **(A)** and GSE14520 **(B)**.

Supplementary Figure 3 The status of HYPERLINK "javascript:;" distinctive biological pathways among CR clusters by GSEA enrichment analysis.

Supplementary Figure 4 GO and KEGG analysis of CR phenotype-associated DEGs. GO **(A)** and KEGG **(B)**.

Supplementary Figure 5 The Venn diagram shows the intersection between the genes involved in the ICGC cohort and CR phenotype-related DEGs **(A)**. The CRsscore in the TCGA cohort. Univariate Cox analyses **(B)**, multivariate Cox analyses **(D)**, nomogram **(C)**, and calibration plot for the nomogram (E).

Supplementary Figure 6 The variation scores of significant HYPERLINK "javascript:;" biological HYPERLINK "javascript:;" processes by ssGSEA analysis among risk subgroups in the ICGC cohort.

Supplementary Figure 7 Relationships between the CRsscore and vital biological processes. Cuproptosis **(A)**, HYPERLINK "javascript:;" glycolysis **(B)**, fatty acid metabolism **(C)**, glutaminolysis **(D)**, PPP **(E)** and the expression of FDX1(F).

Supplementary Figure 8 Relationships between the CRsscore and chemotherapeutic sensitivity **(A–E)**.

## Abbreviations

CR, chromatin regulator; CRsscore, chromatin regulators phenotype-related gene signature; WGCNA, the weighted gene co-expression network analysis; DEGs, differentially expressed genes; HCC, hepatocellular carcinoma; GSVA, gene set variation analysis; LASSO, least absolute shrinkage and selection operator; FAO, fatty acid oxidation; PPP, pentose phosphate pathway; GO, Gene Ontology; KEGG, Kyoto Encyclopedia of Genes and Genomes; OS, overall survival; ROC, received operating characteristic; C-index, Harrell’s concordance index; ssGSEA, single-sample gene set enrichment analysis.
