# Supplementary figures and images for: Characterization of chromatin regulators identified prognosis and heterogeneity in hepatocellular carcinoma
Source: Front Oncol. 2022 Sep 9;12:1002781. doi: 10.3389/fonc.2022.1002781 (PMC9505021; doi:10.3389/fonc.2022.1002781)

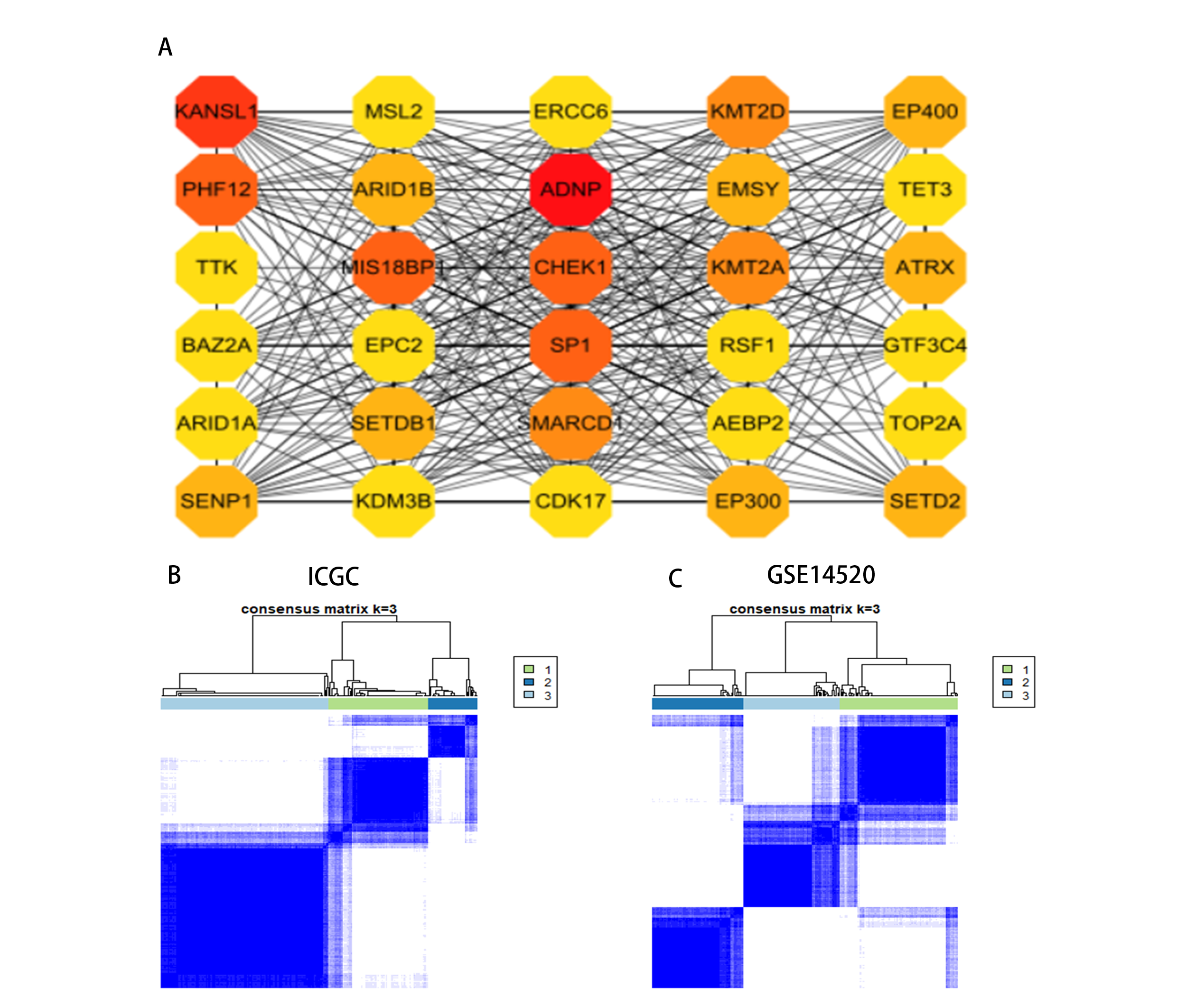

Supplement: Supplementary Figure 1 — The variation scores of significant HYPERLINK "javascript:;" biological HYPERLINK "javascript:;" processes by ssGSEA analysis among risk subgroups in the ICGC cohort. [file Image_1.tif]

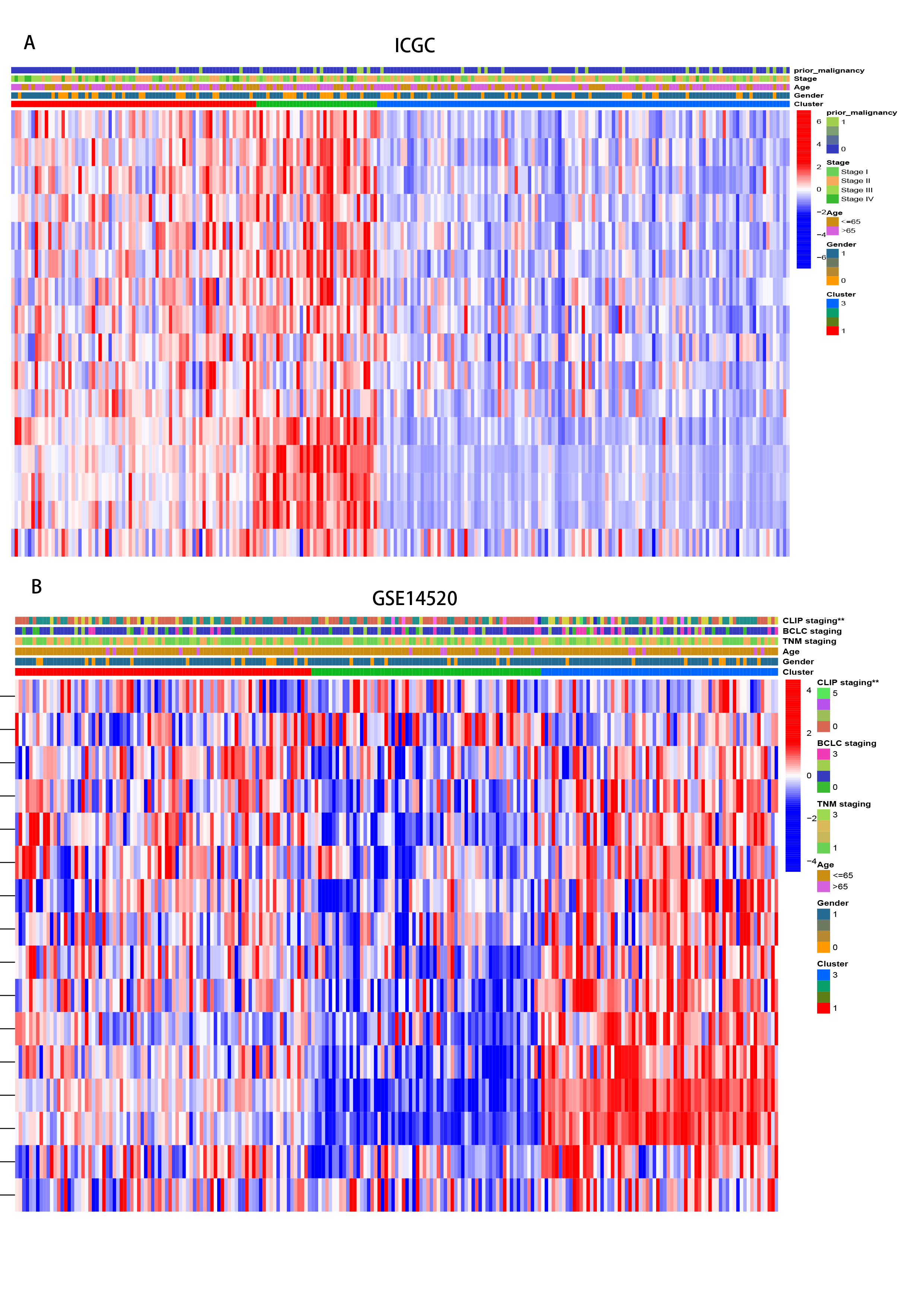

Supplement: Supplementary Figure 2 — Heatmap of the clinicopathological manifestations among CR clusters in the ICGC cohort (A) and GSE14520 (B). [file Image_2.tif]

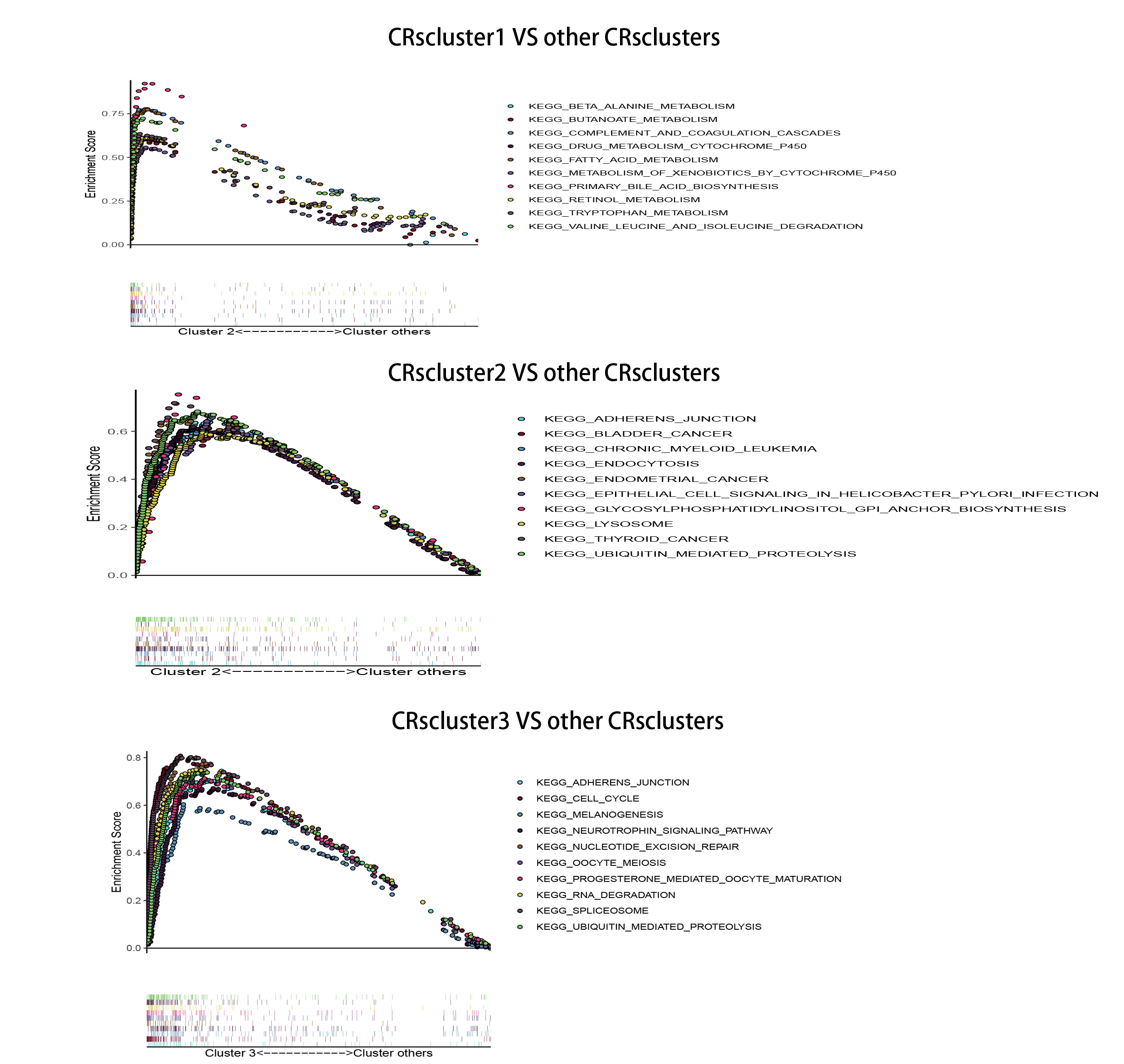

Supplement: Supplementary Figure 3 — The status of HYPERLINK "javascript:;" distinctive biological pathways among CR clusters by GSEA enrichment analysis. [file Image_3.tif]

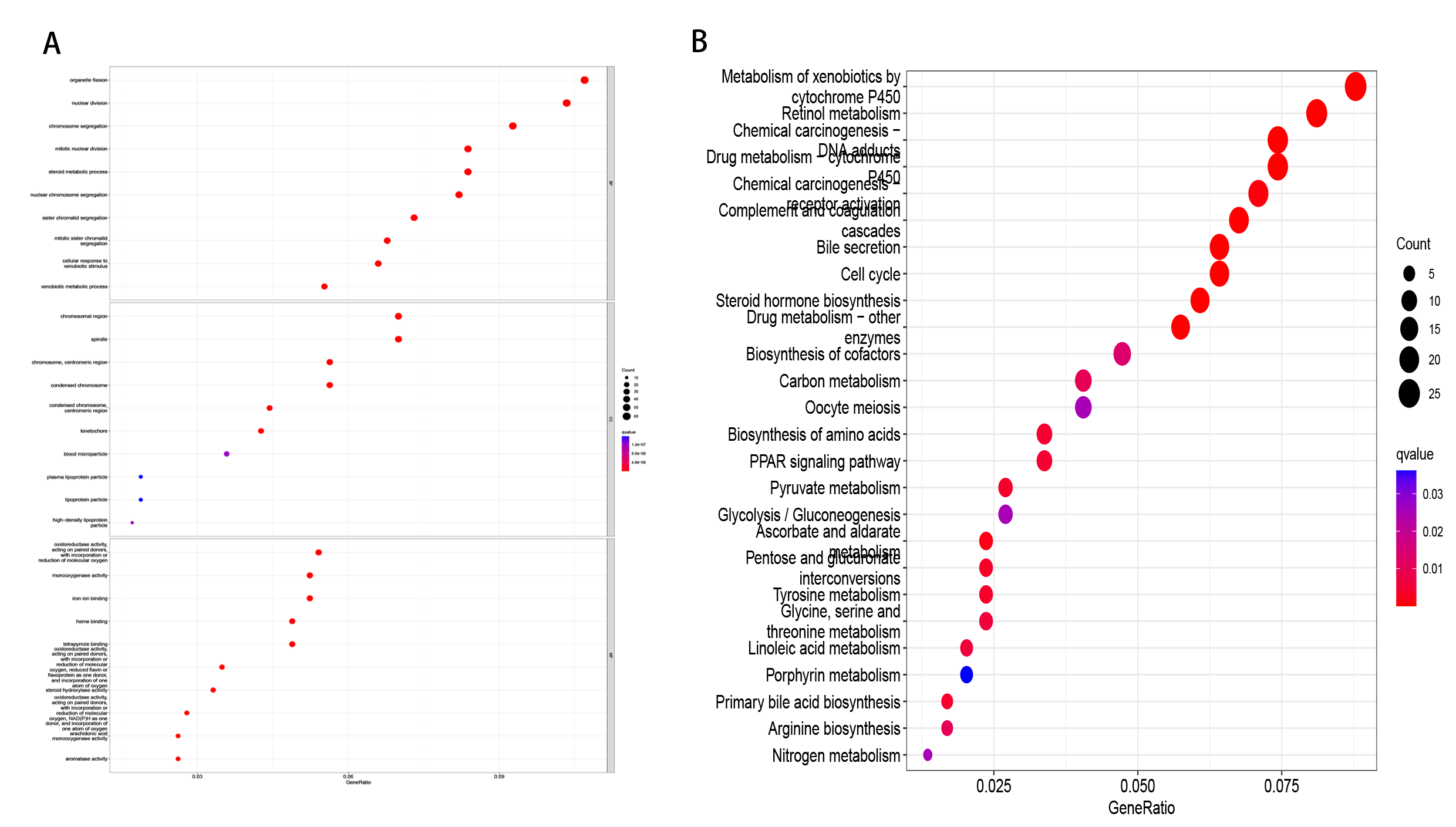

Supplement: Supplementary Figure 4 — GO and KEGG analysis of CR phenotype-associated DEGs. GO (A) and KEGG (B). [file Image_4.tif]

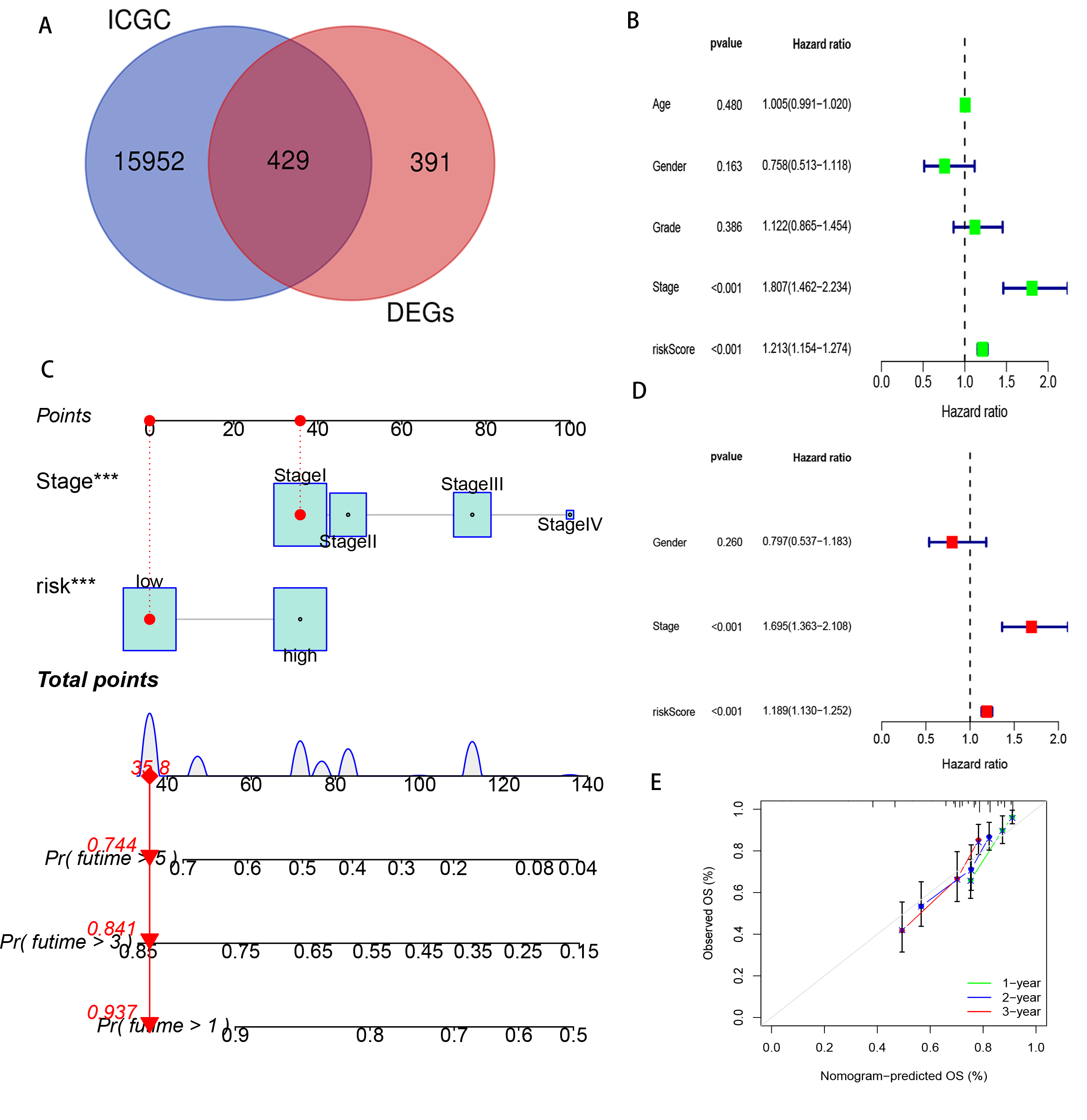

Supplement: Supplementary Figure 5 — The Venn diagram shows the intersection between the genes involved in the ICGC cohort and CR phenotype-related DEGs (A). The CRsscore in the TCGA cohort. Univariate Cox analyses (B), multivariate Cox analyses (D), nomogram (C), and calibration plot for the nomogram (E). [file Image_5.tif]

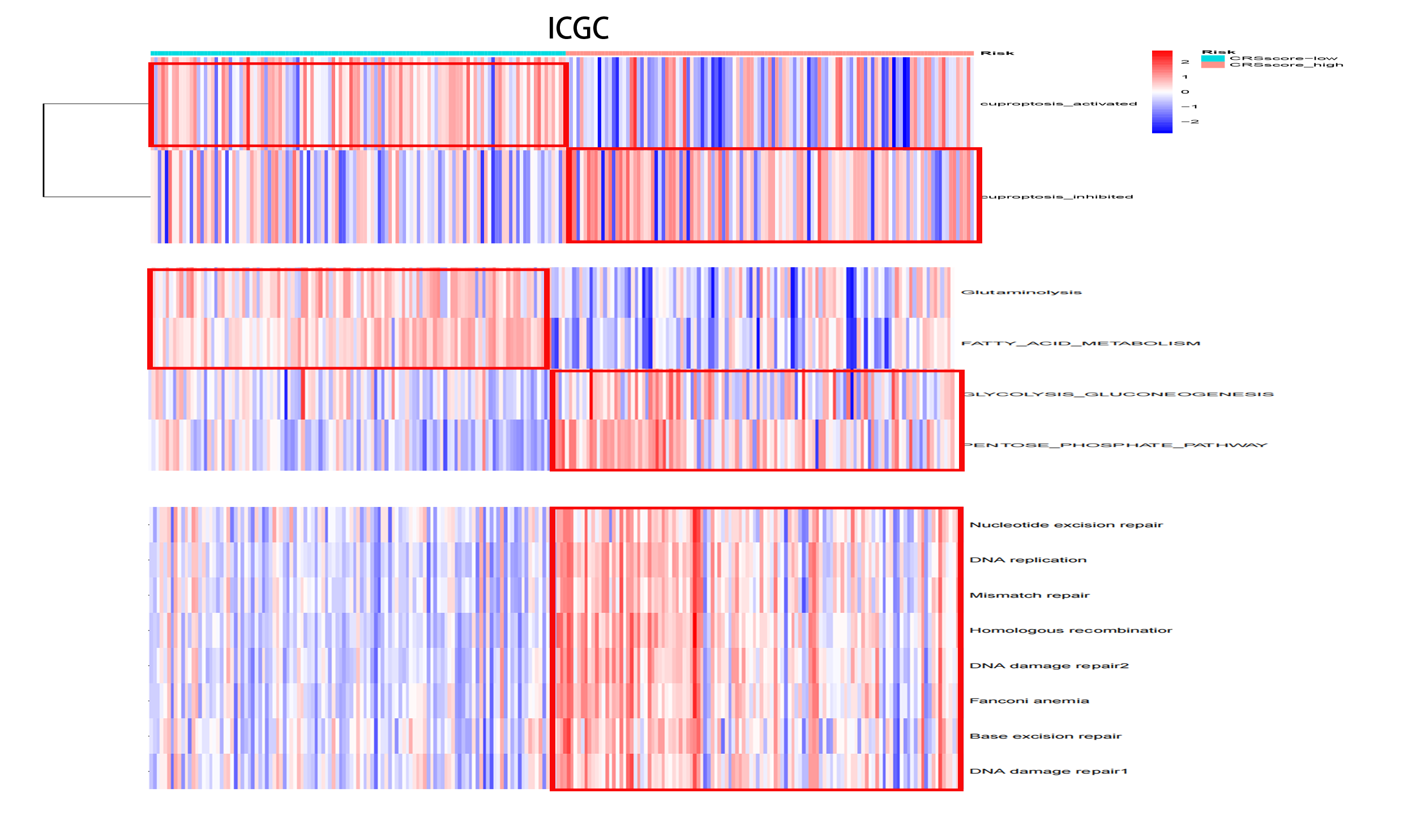

Supplement: Supplementary Figure 6 — The variation scores of significant HYPERLINK "javascript:;" biological HYPERLINK "javascript:;" processes by ssGSEA analysis among risk subgroups in the ICGC cohort. [file Image_6.tif]

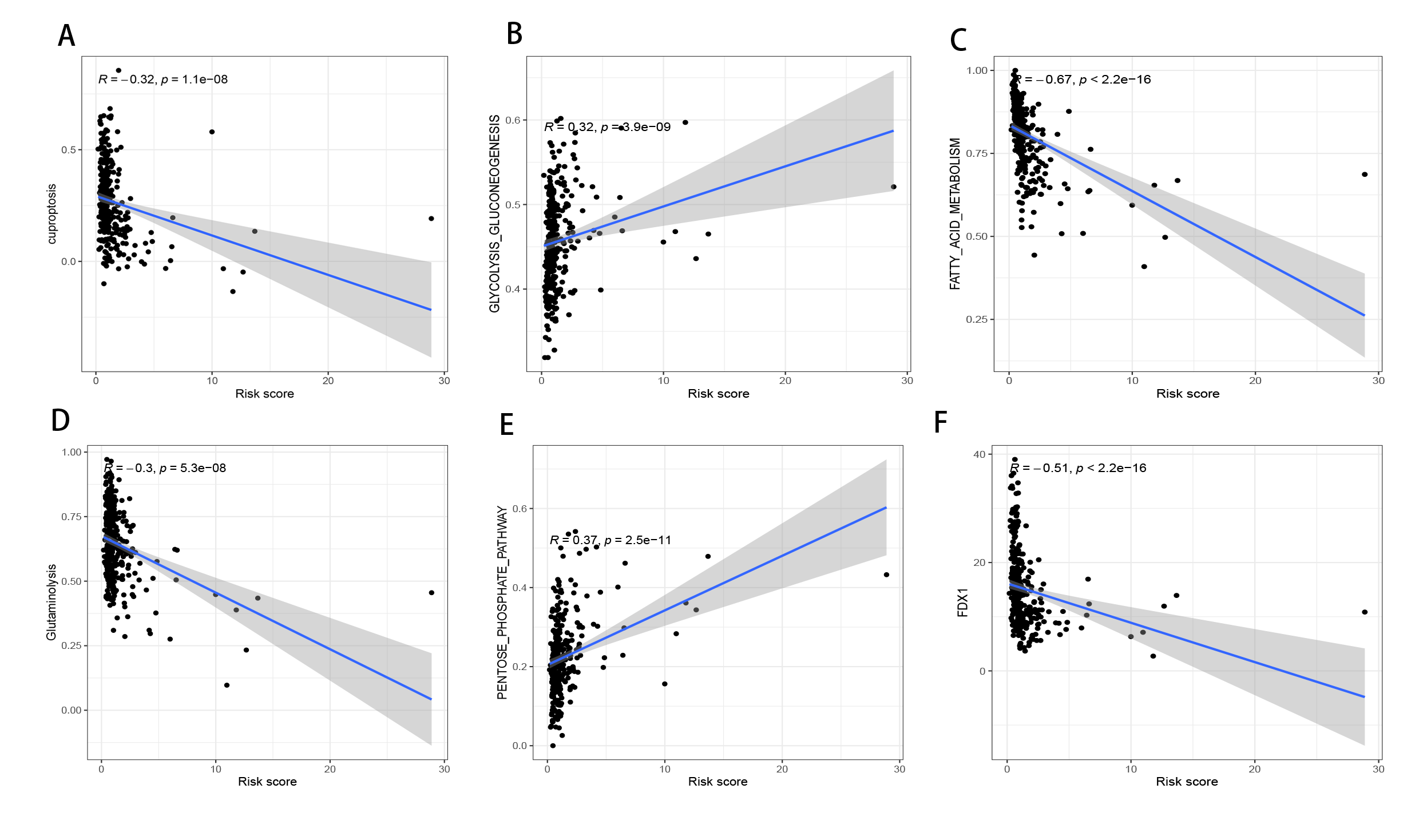

Supplement: Supplementary Figure 7 — Relationships between the CRsscore and vital biological processes. Cuproptosis (A), HYPERLINK "javascript:;" glycolysis (B), fatty acid metabolism (C), glutaminolysis (D), PPP (E) and the expression of FDX1(F). [file Image_7.tif]

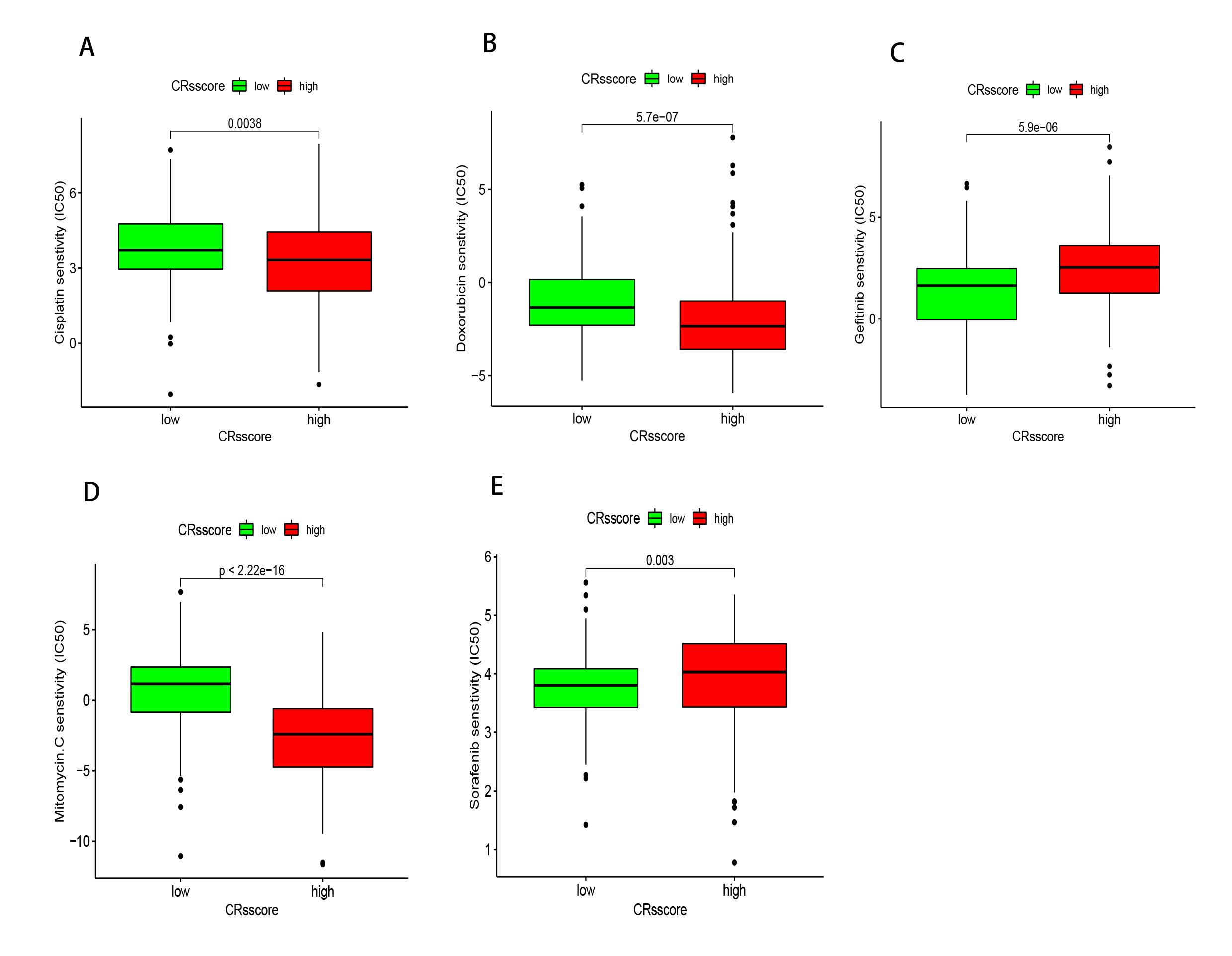

Supplement: Supplementary Figure 8 — Relationships between the CRsscore and chemotherapeutic sensitivity (A–E). [file Image_8.tif]
